# Isoflurane reduces hypoxia/reoxygenation-induced apoptosis and mitochondrial permeability transition in rat primary cultured cardiocytes

**DOI:** 10.1186/1471-2253-14-17

**Published:** 2014-03-10

**Authors:** Wanjun Wu, Xianju Zhou, Ping Liu, Weidong Fei, Li Li, Huifang Yun

**Affiliations:** 1 Department of Anesthesiology, Changzhou No.2 People’s Hospital, the affiliated hospital of Nanjing Medical University, Changzhou 213003, China; 2 Department of Neurology, Changzhou No.2 People’s Hospital, the affiliated hospital of Nanjing Medical University, Changzhou 213003, China

**Keywords:** Isoflurane, Hypoxia/reoxygenation, Cardiocyte, Apoptosis, Mitochondrial permeability transition, ROS

## Abstract

**Background:**

The volatile anesthetic isoflurane protects the heart from hypoxia/reperfusion (H/R) injury. However, it is still incompletely understood whether isoflurane exerts its protective role through preventing mitochondrial permeability transition pore (MPTP) opening.

**Methods:**

Primary cultured cardiocytes were exposed to H/R in the absence or presence of isoflurane. Cell cytotoxicity and apoptosis were detected by MTT assay and TUNEL staining, respectively. MPTP function was monitored by confocal imaging after reoxygenation. ROS production and activation of caspase-3 were determined by fluorescent reader and western blot, respectively.

**Results:**

As compared to the control group, H/R led to significant cell cytotoxicity and apoptosis, while application of isoflurane markedly reversed the effects. Furthermore, isoflurane significantly inhibits the formation of H/R-induced excess ROS production. Finally, isoflurane attenuated the onset of mitochondrial permeability transition pore (MPTP) occurred during hypoxia/reoxygenation, and in turn inhibited activation of caspase-3.

**Conclusions:**

These data indicate that isoflurane has a protective effect on cardiocytes exposed to H/R by reducing excess ROS production, blocking open of MPTP and further reducing apoptosis.

## Background

Hypoxia/reperfusion (H/R)-induced cardiocyte injury is still a clinical problem, particularly after cardiopulmonary bypass (CPB). The main problem in H/R cardiocyte injury is myocardial dysfunction, manifested by arrhythmia and myocardial failure. Volatile anesthetics can protect against reperfusion injury after myocardial ischemia *in vitro* and *in vivo*[1-4]. Isoflurane is one of the drugs most commonly used to maintain the state of general anesthesia. It has been reported that isoflurane preconditioning can mimic ischemia preconditioning to protect against reperfusion injury after myocardial ischemia *in vitro* and *in vivo*[3,4]. However, to date, the mechanisms of isoflurane conditioning remain unclear. Compelling experimental evidence indicates that reactive oxygen species (ROS) play a central role in anesthetic-induced preconditioning (APC). Elevated ROS production during reperfusion enhances Ca^2+^ influx into mitochondria, opens the mitochondrial permeability transition pore (MPTP), and in turn contributes to apoptotic cell death [5]. Previous studies showed that desflurane improves the resistance of the transition pore to Ca^2+^-induced opening after ischemia and reperfusion [6]. In this study, we used primary cultured cardiocytes as an *in vitro* model to investigate the effects of isoflurane on H/R-induced cardiocyte injury.

## Methods

### Experimental animals

All experiments were performed in accordance with the protocols approved by Institutional Animal Care and Use Committee and conformed to the Guide for the Care and Use of Laboratory Animals of Xuzhou Medical College, China. This study was approved by the Animal Ethics Committee of Nanjing Medical University, and was in compliance with the Guide for the Care and Use of Laboratory Animals published by the Chinese National Institute of Health.

### Cardiocyte culture

Neonatal rat Sprague-Dawley hearts (1 ~ 3 days old) were removed under sterile conditions and washed 3 times in phosphate buffered saline (PBS) to remove excess blood cells. The ventricles were minced small pieces and then agitated gently in a solution of 0.125% trypsin. The mixture was centrifuged at 1000 × *g* for 10 min. The supernatant phase was discarded, and the cells were re-suspended in Dulbecco’s modified Eagle’s medium (DMEM) containing 10% fetal bovine serum, 100 U/ml penicillin and 100 μg/ml streptomycin. Isolated cells were cultured in a flask for 1 h at 37°C in order to remove fibroblasts. The suspended cells were diluted to about 5.0 × 10^5^ cells/ml, and seeded in a 6-well plastic plate (2 ml for each well). The cultures were incubated for 72 h in a humidified atmosphere of 5% CO_2_ and 95% air at 37°C.

### Preparation of isoflurane solution

A stock solution of isoflurane dissolved in culture medium was prepared using a modification of the method of Blanck and Thompson [7]. 10 mM isoflurane solution was made by injecting 130 μl liquid isoflurane (Aerrane, Baxter Healthcare Corp., USA) into 100 ml PBS in a 100 ml volumetric flask. The flask was sealed with a glass stopper to exclude all air from the neck of the flask. The flask was wrapped in aluminum foil, and stirred for 24 hours to dissolve isoflurane. Immediately before use, 30 ml of the concentrated stock solution was poured into a 50 ml polypropylene centrifuge tube and vortexed for 5 to 10 seconds to produce the working stock. An aliquot of the working stock was assayed by gas chromatography to determine the concentration of dissolved isoflurane. For treating cell cultures, the working stock was diluted with Dulbecco’s modified Eagle’s medium (DMEM) to produce 0.4 mM isoflurane in solution, equivalent to 2.2% atm isoflurane (equivalent to 1.8 MAC) equilibrated with aqueous medium. The stability of isoflurane in solution was determined by incubating samples of the working stock under identical conditions used in treating cell cultures for a period of 30 minutes. Gas chromatography was used to confirm the concentration of isoflurane.

### Hypoxia and reoxygenation

The hypoxic-reoxygenated cultures were placed within a modular incubator chamber (Thermo, USA), filled with 1% O_2_, 5% CO_2_, and balanced N_2_ for 3 h, then 21% oxygen for 2 h. For hypoxia, the culture media were replaced by a modified Tyrode’s solution [8] (in mM/l: 136.9 NaCl, 2.68 KCl, 8.1 Na_2_HPO4 · 12 H_2_O, 1.47 KH_2_PO4, 0.9 CaCl_2_, and 0.49 MgCl_2_ · 6 H_2_O; pH 7.4). Control cultures were continuously incubated in 5% CO_2_ and 95% air at 37°C.

Cardiocytes were randomly divided into 4 groups: 1) Control group, cardiocytes were incubated at 37°C in a humidified atmosphere of 5% CO2 and 95% air for 5 hours; 2) hypoxia/reoxygenation group, cardiocytes were exposed to hypoxia (1% O2 and 5% CO2 ) for 3 hr followed by 2 hr reoxygenation (95% air and 5% CO2 ); 3) Isoflurane with hypoxia/reoxygenation group, 3 hr hypoxia followed by 2 hr reoxygenation, isoflurane (0.4mΜ) solution was added in medium during hypoxia; 4) Isoflurane without hypoxia/reoxygenation group, cardiocytes were incubated under a condition similar to control group in the presence of isoflurane. Calcein imaging was performed after hypoxia for 3 hr followed by 50 min reoxygenation due to the better imaging effect at that time.

### MTT assay

The cell suspensions were diluted to a concentration of ~ 5 × 10^4^ cells/ml. A 100 μl aliquot containing ~5 × 10^3^ cells was added immediately to each well of a 96-well flat bottom microplate in sextuplet. After a period of 72 h incubation at 37°C in 5% CO_2_, the cultures were subjected to hypoxia/reoxygenation. Isoflurane solution (Baxter, USA) was added to the wells at final concentration of 0.4mΜ. Cardiocyte Cytotoxicity was assayed with a MTT (3-4,5-Dimethylthiazol-2-yl)-2,5-diphenyltetrazolium bromide) cell proliferation and cytotoxicity assay kit (Beyotime, Shanghai, China). The absorbance was determined at a wave length of 492 nm using an ELISA reader as the cardiocyte-cytotoxicity value [9].

### TUNEL labeling

Terminal deoxynucleotidyl transferase-mediated dUTP nick-end labeling (TUNEL) was used to assess cardiocyte apoptosis in this study [10]. The cultures were analyzed for apoptotic DNA fragmentation by using the One Step TUNEL Apoptosis Assay Kit (Beyotime, Shanghai, China). dUTP conjugated with fluorescein isothiocyanate (FITC) labels the 3'-OH terminal of the DNA strand breaks, which was catalyzed by Terminal deoxynucleotidyl transferase. Cardiocytes incubated in 6-well microplates for 72 h were washed with PBS and fixed in 4% paraformaldehyde for 30 min. After washed with PBS, the cells were incubated with cold PBS containing 0.1% Triton X-100 for 2 min in a dark condition. Following PBS wash, the cells were incubated with a TUNEL reaction buffer for 1 h at 37°C in a humidified chamber away from light. The cultures were finally washed with PBS three times, Fluorescence was detected with emission wavelength at 530 nm and excitation wavelength at 485 nm using a laser scanning confocal microscope (LSCM, Leica, Germany), and the fluorescence intensity of FITC was analyzed with Image-Pro plus 6.0 software.

### Calcein imaging by confocal microscopy

Calcein-AM is permeable to the mitochondria, and is de-esterified and trapped in the matrix space in its free fluorescent form (calcein). Therefore, after cold ester loading/warm incubation, most calcein-AM localized exclusively to mitochondria. Calcein leaks from the mitochondria when MPTP opens. As a result, calcein fluorescence decreases in the mitochrondria, and redistributes to the cytosol and nucleus [11]. Myocytes were loaded with Calcein-AM (1 μM) for 30 min at 4°C in HEPES-buffered Dulbecco's modified eagle medium (DMEM, 20 mM HEPES) containing 10% fetal calf serum at pH 7.4. After cold loading, cells were further incubated for 5 h at 37°C in DMEM without serum. Prior to mounting on the microscope, cells were washed twice with PBS [12]. Fluorescence was detected with emission wavelength at 515 nm and excitation wavelength at 490 nm using a laser scanning confocal microscope (LSCM, Leica, Germany).

### Western blot

After treatment, samples were collected in 40 ml of SDS sample buffer (10 mM Tris-HCl buffer, pH 6.8, 2% sodium dodecylsulfate, 10% glycerol, 0.01% bromophenol blue and 5% β-mercaptoethanol), and boiled at 100°C for 5 min. The extracts were separated by 20% SDS-PAGE, transferred to nitrocellulose membranes, and blocked with 5% non-fat milk in PBS-T (PBS, 0.1% Triton X-100) for 1 hr at room temperature. The membranes were then incubated with primary antibodies against cleaved caspase-3 (1:1000, Cell signaling) in PBS-T overnight at 4°C. Following incubation with horseradish peroxidase-conjugated goat anti-rabbit antibodies (1:5000; Pierce, Rockford, IL, USA), the signals were detected by the chemiluminescence method (SuperSignal West Pico, Pierce, Rockford, IL, USA). For the purpose of normalization, the membranes were stripped, and re-probed with antibodies against β-actin (1:10,000; Sigma, St Louis, MO, USA). Several exposures were used to obtain signals in the linear range. The bands were quantified by using Image-Pro plus 6.0 software.

### Measurement of ROS

Measurement of ROS production was performed as described previously with slight modifications [13]. Briefly, cells growing in 6 well plates at a density of about 5 × 10^5^/well, in the presence or absence of isoflurane preconditioning, were loaded with 5 μM DCFH-DA (Molecular Probe, Eugene, USA) diluted in PBS buffer and incubated for 30 minutes. DCFH-DA penetrated into the cytoplasm, and was converted to 2’,7’-dichlorofluorescin (DCFH) by the hydroxylation of cellular esterase. Oxidation of DCFH by hydrogen peroxide and hydroxyl radicals generates a fluorescent product, 2’,7’-dichlorofluorescein (DCF). The intensity of DCF fluorescence was determined by a Chameleon multi-label plate reader (Hidex Personal Life Science, Finland), with an excitation wavelength of 450 nm and an emission wavelength of 535 nm. The relative fluorescence intensity was expressed as a percentage of the control group.

### Statistical analysis

Data were expressed as mean ± SD. One-way ANOVA followed by the *post hoc* (SNK) test was used to determine significant differences among all groups. *P* values less than 0.05 were considered to be significant.

## Results

### Isoflurane attenuates hypoxia/reperfusion-induced cell cytotoxicity

**Figure 1:**
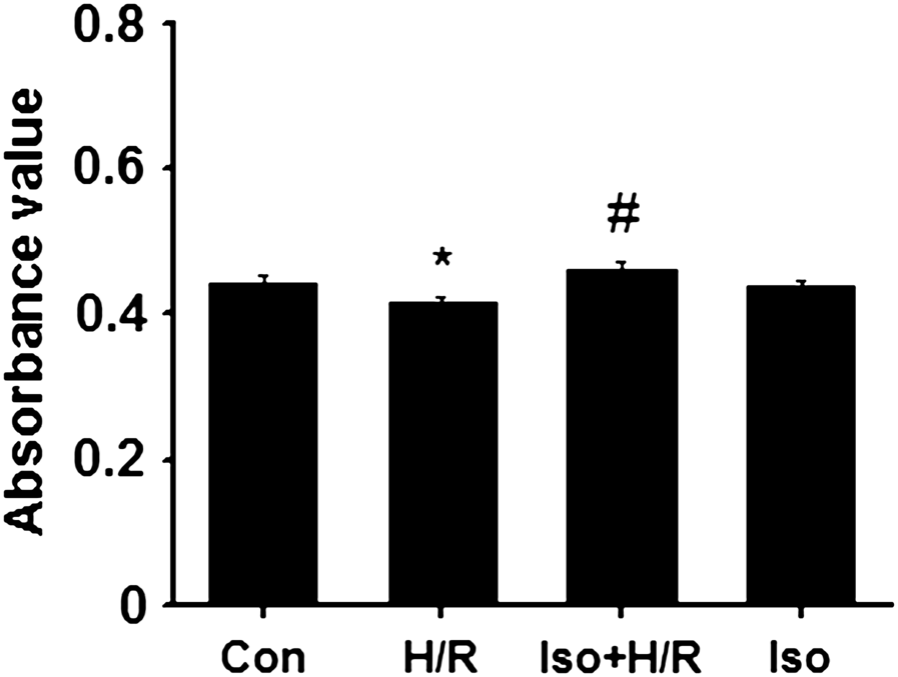
**Viability value of each group (n = 6).** *p < 0.01, compared to the control group; # p < 0.01, compared to the H/R group. Con, control; H/R, hypoxia/reoxygenation; Iso + H/R, isoflurane plus H/R; Iso, isoflurane alone. The concentration of isoflurane was 0.4 mM.

MTT assay was used to detect the cardiocyte cytotoxicity reaction. The absorbance analyzed at 492 nm with ELISA reader is shown in Figure 1. As compared to the control group, hypoxia/reperfusion significantly reduced the value of absorbance (0.417 ± 0.006 versus 0.442 ± 0.010, p < 0.01), indicating its induction of cytotoxicity. There was a significant difference in absorbance between hypoxia/reperfusion and isoflurane group (0.417 ± 0.006 versus 0.462 ± 0.011, p < 0.01), suggesting that addition of isoflurane markedly reversed the toxic effect of hypoxia/reperfusion. Additionally, the isoflurane alone group (without hypoxia/reperfusion) did not display a significant difference as compared to the control group (0.437 ± 0.008 versus 0.442 ± 0.010, p < 0.05). These results suggest that isoflurane attenuates cell cytotoxicity triggered by hypoxia/reperfusion.

### Isoflurane protects against hypoxia/reperfusion-induced cardiocyte apoptosis

**Figure 2:**
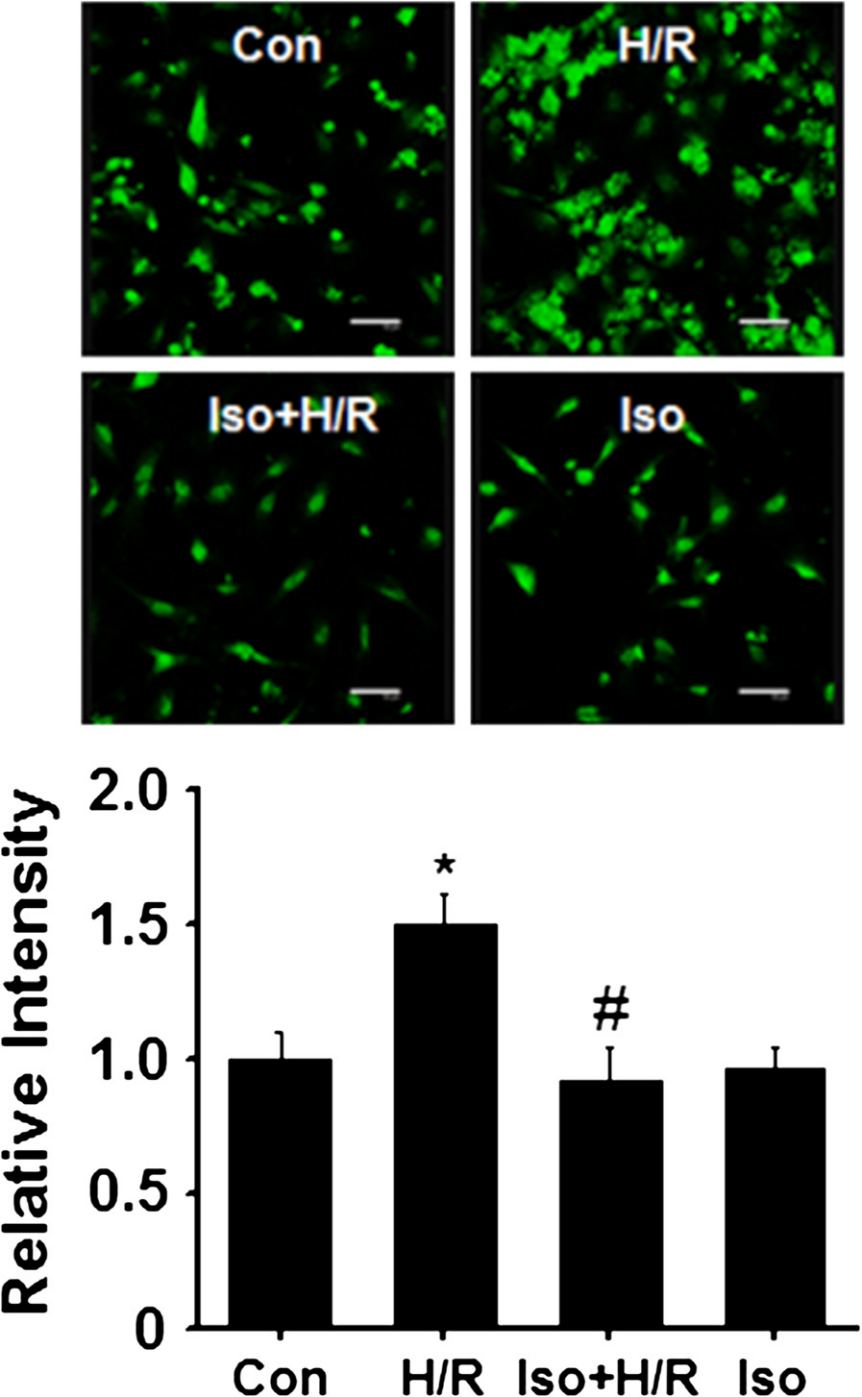
**Effects of isoflurane on H/R-induced apoptosis shown by TUNEL labeling.** Top panel, representative images; Bottom panel, quantification of fluorescence intensity (cells were randomly selected from different areas; each value was from three separate experiments). Con, control (n = 34); H/R, hypoxia/reoxygenation (n = 38); Iso + H/R, isoflurane plus H/R (n = 40); Iso, isoflurane alone (n = 36). *p < 0.01, compared to the control group; # p < 0.01, compared to the H/R group. Scale bar, 50 μm.

It is known that hypoxia/reperfusion can induce significant cardiocyte apoptosis, thus we further wanted to know if isoflurane prevent cardiocytes from the apoptosis. As shown in Figure 2, by using TUNEL staining, exposure of cardiocytes to hypoxia/reoxygenation caused a significant increase in apoptosis as compared with the control group (p < 0.01). Additionally, isoflurane itself did not lead to any significant change in apoptosis (Figure 2). Together, in agreement with cytotoxicity data, addition of isoflurane to cultured cardiocytes greatly attenuated hypoxia/reoxygenation-induced cardiocyte apoptosis.

### Isoflurane prevents opening of MPTP

**Figure 3:**
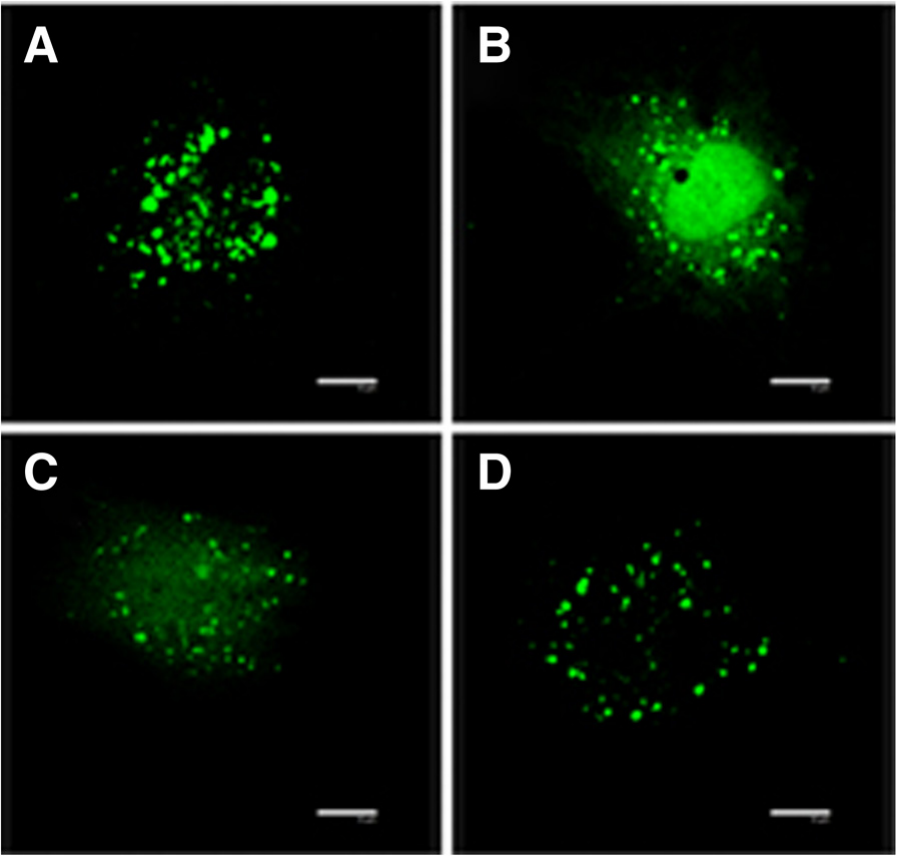
**Isoflurane prevents MPTP opening of cultured cardiocytes exposed to hypoxia/reperfusion.** Representative images of single cardiomyocytes from the four different groups are shown. **(A)** Con, control; **(B)** H/R, hypoxia/reoxygenation; **(C)** Iso + H/R, isoflurane plus H/R; **(D)** Iso, isoflurane alone. The fluorescent dots indicate mitochondria; the arrow indicates the nucleus. Scale bar, 10 μm.

Previous studies indicate that opening of the mitochondrial permeability transition pore (MPTP) is associated with cardiocyte apoptosis occurred during cardiocyte reperfusion injury, thus we wondered if isoflurane protects cardiocytes by preventing MPTP opening. To reveal directly opening of the MPTP after reperfusion, we imaged calcein by confocal microscopy. As shown in Figure 3A, in the cardiocyte of the control group, when 30 minutes of cold ester loading was followed by 5 hours of warm incubation, fluorophore loading appeared to be exclusively confined to the mitochondria. In contrast, when cardiocytes were subjected to hypoxia/reoxygenation at pH 7.4 (Figure 3B), calcein fluorescence was released from mitochondria and redistributed to the cytosol and nucleus. When isoflurane was added during hypoxia/reoxgenation (Figure 3C), the redistribution were significantly attenuated. Isoflurane alone did not change the redistribution (Figure 3D). These findings suggest that isoflurane prevents MPTP opening during hypoxia/reoxygenation.

### Isoflurane inhibits hypoxia/reperfusion-induced activation of caspase-3

**Figure 4:**
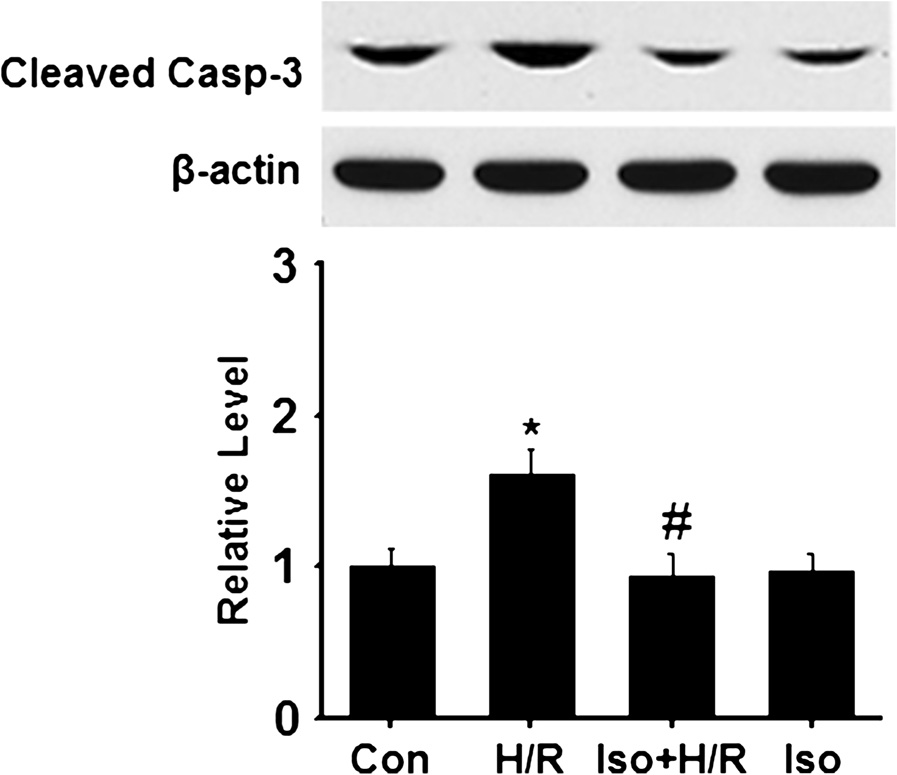
**Isoflurane inhibits activation of caspase-3.** Samples were collected after cardiocytes were differently treated. The level of cleaved caspase-3 and β-actin were examined by Western blot. The signal of cleaved caspase-3 Asp175 was quantified and normalized to β-actin. Top panel, representative blots; Bottom panel, quantification of relative activated caspase-3 level to the control group. Data were collected from three independent preparations for each group. *p < 0.01, compared to the control group; # p < 0.01, compared to the H/R group. Con, control; H/R, hypoxia/reoxygenation; Iso + H/R, isoflurane plus H/R; Iso, isoflurane alone.

MPTP opening will initiate the apoptotic pathways. Downstream activation of caspase-3, a key enzyme to execution stage of the apoptotic pathway, plays a vital role in apoptosis. Next, we examined activation of caspase-3 following hypoxia/reperfusion by western blot. As seen in Figure 4, exposure of cardiocytes to hypoxia/reoxygenation significantly increased endogenous level of the large fragment (cleaved caspase-3 at Asp175) of activated caspase-3. In the presence of isoflurane, the endogenous level of the cleaved caspase-3 was greatly down-regulated. Isoflurane itself appeared not to affect the activation of caspase-3 (Figure 4). These results suggest that isoflurane inhibits activation of caspase-3 by preventing MPTP opening.

### Isoflurane reduces hypoxia/reperfusion-induced ROS production

**Figure 5:**
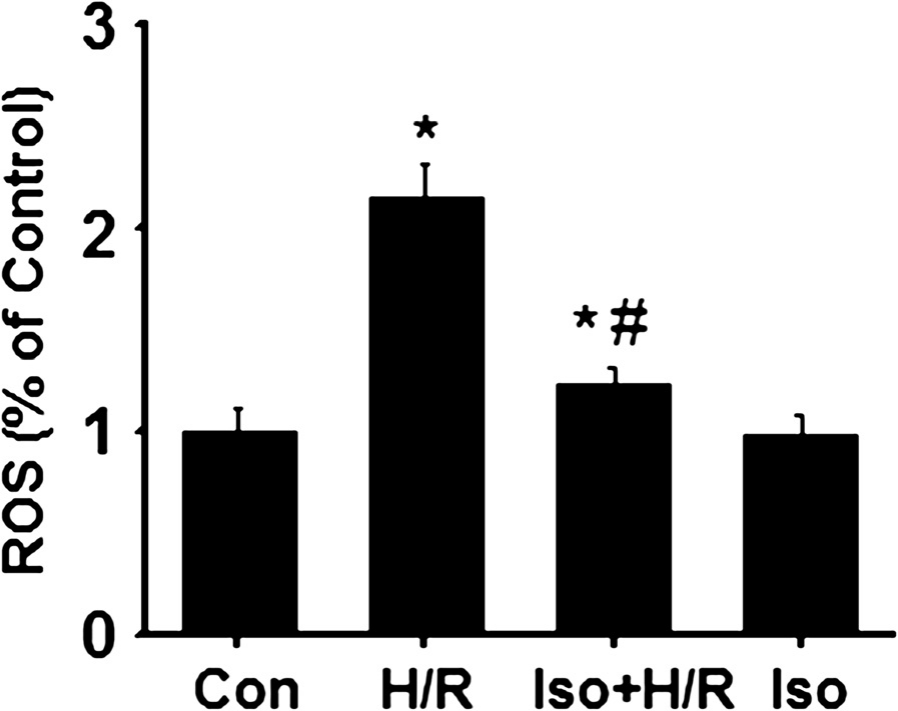
**Isoflurane prevent the formation of H/R-induced ROS production.** Samples were collected after cardiocytes were differently treated and loaded with 5 μM DCFH-DA. The intensity of DCF fluorescence was determined by a Chameleon multilabel plate reader. The relative fluorescence intensity was expressed as a percentage of the control group. Data were collected from three independent preparations for each group. *p < 0.01, compared to the control group; # p < 0.01, compared to the H/R group. Con, control; H/R, hypoxia/reoxygenation; Iso + H/R, isoflurane plus H/R; Iso, isoflurane alone.

It is well-known that excess ROS production is regarded as an initial cause of H/R injury [14]. As major sites of ROS production, the mitochondria play a key role in ROS-related injury. We wondered if isoflurane pretreatment prevents the formation of reactive oxygen species (ROS) production induced by H/R. As many studies showed, we observed that H/R triggered a significant increase in the ROS level when compared to the control group (Figure 5). Isoflurane preconditioning substantially decreased the level of ROS production (Figure 5). Interestingly, the level was still higher than the control group, suggesting that the proper ROS production may improve cardiocyte survival. Additionally, we also observed that isoflurane at 0.4 mM itself did not influence the formation of ROS production (Figure 5). Together, the results point to the inhibitory role of isoflurane in H/R-induced ROS formation.

## Discussion

This study was designed to investigate the effects of isoflurane on cultured cardiocytes exposure to H/R. Our data suggest that: 1) isoflurane pretreatment significantly attenuates the cardiocyte apoptosis induced by H/R; 2) Isoflurane pretreatment prevents formation of excess ROS production, attenuates MPTP opening, and in turn inhibits activation of caspase-3.

Previous studies showed that volatile anesthetics isoflurane preconditioning can protect against reperfusion injury following myocardial ischemia *in vitro* and *in vivo*[3,4]. Compared to other volatile anesthetics, for example sevoflurane and desflurane, isoflurane showed a more powerful potency to reduce myocardial infarct size in experimental animals with preconditioning paradigm [15]. In line with our *in vitro* results, isoflurane-treated mice which were subjected to ischemia followed by 2 weeks of reperfusion exhibited reduction of expression of cleaved caspase-3 and TUNEL staining [16].

The key roles of mitochondria in isoflurane-induced cardioprotection are well established. The potential mechanisms involves PKC translocation to mitochondria, phosphorylation of aldehyde dehydrogenase 2 (ALDH2), mitochondrial depolarization, matrix acidification, MPTP, mitochondrial Ca^2+^ homeostasis, mitochondrial K_ATP_ channels and so on [17-19]. Opening of MPTP is related to mitochondrial damage, and further triggers apoptotic pathways. In this study, we directly observed the inhibitory role of isoflurane pretreatment in H/R-induced opening of MPTP by the confocal imaging, supporting previous research results [20]. Because the mitochondria are major sites of ROS generation, we further observed that H/R-induced excess ROS production was prevented in the presence of isoflurane, supporting a link of excess ROS formation as an upstream signal to MPTP opening. Intriguingly, the protective role of isoflurane appeared to be associated with a proper level of ROS generation. Consistently, increased ROS production (compared to the control experiments) following isoflurane inhalation caused a tolerance against myocardial infarction *in vivo*[21,22]. Together, the beneficial effect of isoflurane is at least in part achieved by adjusting excess ROS production to a proper level during H/R.

Opening of the MPTP is related to swelling and uncoupling of mitochondria as occurs in damaged tissues by reperfusion injury [23]. The swelling of the mitochondria appears to be sufficient to activate caspases and thus induce apoptosis [24]. In our study, we focused on the effect of isoflurane on opening of MPTP, and clearly observed that isoflurane decreased the opening of MPTP, and inhibited activation of its downstream caspase-3. In agreement with our results, a previous *in vivo* report showed that isoflurane post-conditioning prevented opening of MPTP, and as a result protected mouse hearts from reperfusion injury [25]. Though using a different paradigm (post-conditioning versus preconditioning), common mechanisms may be shared. In support, another report showed a key role of cytochrome c release from the mitochondria in the pathogenesis of ischemia/reperfusion injury by examining cytochrome c in the mitochondria and cytosol, suggesting that the myocardioprotective effects of isoflurane preconditioning were associated with inhibition of cytochrome c loss from mitochondria [26]. Therefore, preventing MPTP opening is central to myocardioprotection induced by isoflurane pretreatment.

## Conclusions

The volatile anesthetic isoflurane pretreatment protects against cardiocyte ischemia and reperfusion injury by multiple mechanisms. Our findings suggest that isoflurane has protective effects on cardiocytes exposed to H/R by preventing ROS formation, blocking MPTP opening and further reducing activation of apoptotic protein caspase-3.

## Competing interests

The authors declare that they have no competing interests.

## Authors’ contributions

WW: performed the experiments, collected and analyzed data, and wrote the manuscript. XZ: collected and analyzed data, and wrote the manuscript. PL WF and LL: collected and analyzed data. HY: designed and supervised this study, analyzed and explained data, as well as wrote the manuscript. All authors read and approved the final manuscript.

## Authors’ information

This work was supported by Department of Anesthesiology, Changzhou NO.2 People’s Hospital.

## Pre-publication history

The pre-publication history for this paper can be accessed here:

http://www.biomedcentral.com/1471-2253/14/17/prepub
